# Reinvestigation of the Oxidative Folding Pathways of Hen Egg White Lysozyme: Switching of the Major Pathways by Temperature Control

**DOI:** 10.3390/ijms140713194

**Published:** 2013-06-26

**Authors:** Kenta Arai, Wataru Shibagaki, Reina Shinozaki, Michio Iwaoka

**Affiliations:** Department of Chemistry, School of Science, Tokai University, Kitakaname, Hiratsuka-shi, Kanagawa 259-1292, Japan; E-Mails: k-arai4470@tokai-u.jp (K.A.); moru-arany0618@ezweb.ne.jp (W.S.); 3bskm003@mail.tokai-u.jp (R.S.)

**Keywords:** lysozyme, oxidative protein folding, temperature effect, selenoxide, reduction pulse, oxidation pulse

## Abstract

It has been well established that in the oxidative folding of hen egg white lysozyme (HEL), which has four SS linkages in the native state (N), three des intermediates, *i.e.*, des[76–94], des[64–80], and des [6–127], are populated at 20 °C and N is dominantly formed by the oxidation of des[64–80] and des[6–127]. To elucidate the temperature effects, the oxidative folding pathways of HEL were reinvestigated at 5–45 °C in the presence of 2 M urea at pH 8.0 by using a selenoxide reagent, DHS^ox^. When reduced HEL was reacted with 1–4 equivalents of DHS^ox^, 1S, 2S, 3S, and 4S intermediate ensembles with 1–4 SS linkages, respectively, were produced within 1 min. After the oxidation, 3S was slowly converted to the des intermediates with formation of the native structures through SS rearrangement. At 5 °C, des[76–94] was populated in the largest amount, but the oxidation to N was slower than that of des[64–80] and des[6–127]. At 35 °C, on the other hand, des[64–80] and des[6–127] were no longer stable, and only des[76–94] was populated. The results suggested that the major folding pathways of HEL can be switched from one to the other by temperature control.

## 1. Introduction

Understanding oxidative folding pathways of SS-containing proteins is important for elucidation of the processes that occur in the endoplasmic reticulum (ER) of eukaryotes as well as in bacterial cells. In the ER, a polypeptide chain synthesized at the ribosome folds into the specific three-dimensional conformation through rapid formation of the native SS linkages under physiological conditions [1,2]. In *in vitro* experiments, however, different pH or temperature conditions are usually applied due to the limited reactivity of available redox reagents. Under such conditions, it would be possible that the major folding pathways revealed *in vitro* are not directly applicable to the *in vivo* pathways. Recently, the folding conditions close to *in vivo*, such as utilization of molecular chaperones [3–5], have been actively examined in *in vitro* studies. In this paper, we have reinvestigated the oxidative folding pathways of hen egg white lysozyme (HEL), a classical model for SS-coupled folding study, by using a water-soluble selenoxide reagent, DHS^ox^, at various temperatures. It was found that the pathways toward N, which are dominant at low temperatures, become hindered and another pathway, which is hindered at low temperatures, becomes the only effective pathway at the temperature near physiological conditions.

**Figure 1:**
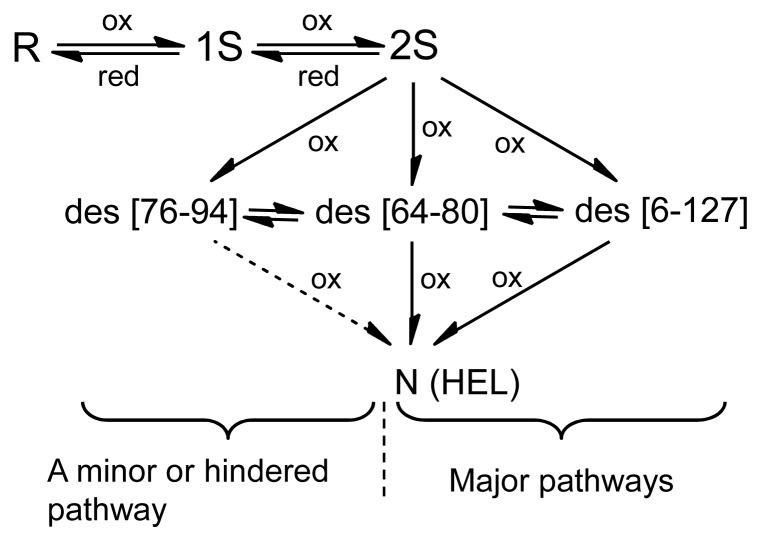
Oxidative folding pathways of HEL at 20 °C and pH 8.5 in the presence of 2 M urea by using 1.0 mM GSH/0.2 mM GSSG as a redox buffer. This figure was modified from the literature [7,8].

HEL (14 kDa) [6] is a lytic enzyme, which hydrolyzes the β-1,4 glycosidic bond between *N*-acetylglucosamine and *N*-acetylmuramic acid in the cell wall of eubacterium. The native structure of HEL is stabilized by four SS linkages (Cys76–Cys94, Cys64–Cys80, Cys6–Cys127, Cys30–Cys115). The oxidative folding pathways have been extensively studied, and the most probable pathways were established by van den Berg *et al.*, as shown in Figure 1[7,8]. When the oxidative folding of reduced HEL (R) having no SS bond was initiated in a pH 8.5 redox buffer solution containing 1.0 mM GSH/0.2 mM GSSG and 2 M urea at 20 °C, the ensembles of oxidized species 1S and 2S having one and two SS bonds, respectively, were gradually generated from R with a time constant of 200~300 s, accompanying hydrophobic collapse burying the tryptophan residues. The generated 2S was then slowly oxidized to three des intermediates, *i.e.*, des[76–94], des[64–80], and des[6–127], which are the key folding intermediates with a native-like conformation stabilized by the three native SS linkages. Finally, by oxidation of the two Cys residues remaining in the des intermediates, the native state (N) was regenerated. While the oxidation of des[64–80] and des[6–127] to N share the major folding pathways of HEL, the oxidation of des[76–94] is hindered and occurs only with a long reaction time or in the presence of protein disulfide isomerase (PDI) probably through the other two des intermediates [8]. However, the folding scheme of Figure 1 left some questions as to the oxidation process from 2S to the des intermediates (*i.e.*, what is the direct precursor of the des intermediates?) and the major folding pathways at higher temperatures close to physiological conditions.

We recently developed a new analytical strategy, *i.e.*, the temporary separation of the SS rearrangement process from the SS formation process by using *trans*-3,4-dihydroxyselenolane oxide (DHS^ox^) as a strong oxidant, for the study of the oxidative folding pathways of SS-containing proteins [9]. DHS^ox^ enables rapid, irreversible, and stoichiometric SS formation in various polythiol peptides in an aqueous medium [10]. For example, when this analytical method was applied to the oxidative folding of bovine pancreatic RNase A having four SS linkages in the native state, the SS formation and SS rearrangement phases were clearly separated from each other, and the oxidative folding pathways could be easily characterized [11,12]. Encouraged by the success, we have applied herein the DHS^ox^ strategy to HEL to characterize the precursors of the des intermediates as well as to identify the major folding pathways at various temperatures.

## 2. Results

### 2.1. Stoichiometric SS Formation of R Using DHS^ox^

**Figure 2:**
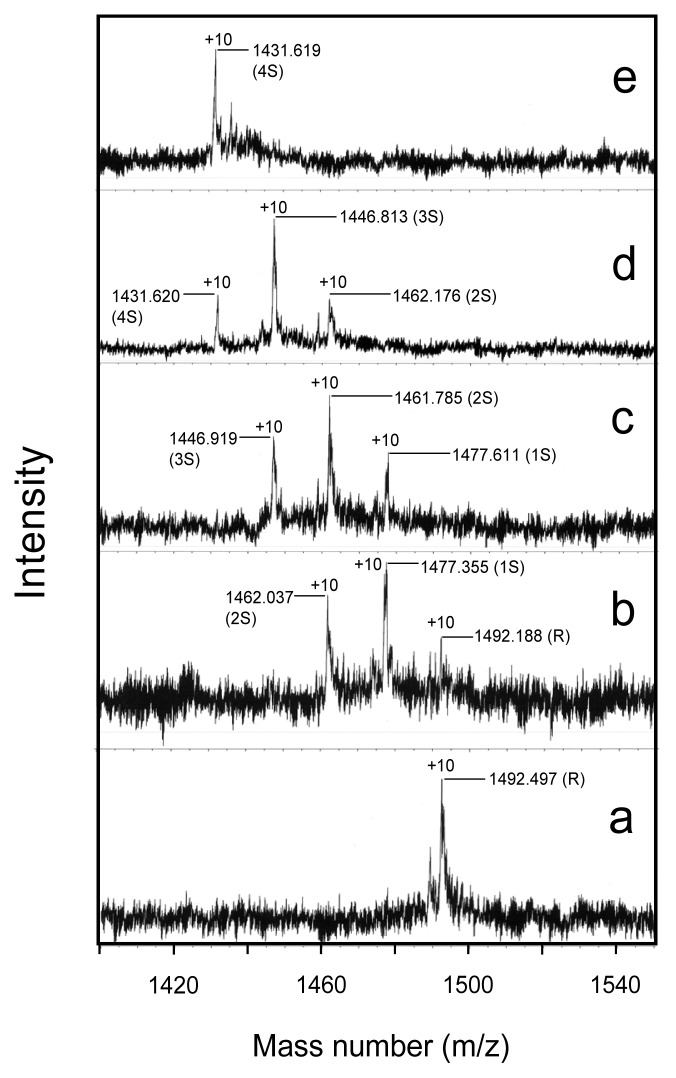
Expanded ESI(+) mass spectra of the sample solutions (with AEMTS blocking) obtained from the short-term oxidative folding of HEL at 25 °C and pH 8.0 in the presence of 4 M urea. (**a**) Reduced HEL ([R]_0_ = 103 μM); (**b**) 1 eq DHS^ox^; (**c**) 2 eq DHS^ox^; (**d**) 3 eq DHS^ox^; and (**e**) 4 eq DHS^ox^ were reacted with R for 1 min.

ESI mass spectra of the sample solutions obtained when R was reacted with 1, 2, 3, and 4 equivalents of DHS^ox^ for 1 min in the presence of 4 M urea are shown in Figure 2. The spectra are expanded in the region of the most intense peak corresponding to MH_10_^10+^. The symbols 1S, 2S, 3S, and 4S indicate intermediate ensembles of the oxidized species having random (or statistic) one, two, three, and four SS bonds, respectively. The high concentration of urea was applied in this oxidation reaction because the unfolded states of HEL have strong propensity to form the aggregates. It should be noted, however, that the same oxidation reaction can be carried out in 2 M urea to yield the products, which are similar to those obtained in 4 M urea as judged by the peak profiles of the HPLC chromatograms (data not shown).

Since modification of the folding intermediates by AEMTS caused an increment of the molecular mass by 76 Da per one free SH group [9], the number of the SS bonds for each intermediate could be determined based on the observed mass number (see Table S1). The results show that the major SS components were 1S, 2S, 3S, and 4S, respectively, when 1, 2, 3, and 4 equivalents of DHS^ox^ were reacted, suggesting that the SS formation was completed stoichiometrically. Indeed, according to Ellman’s assay, the amount of the SH groups remaining in the intermediate mixture was quantitatively decreased with the amounts of DHS^ox^ added (see Figure S1). Thus, the SS formation proceeded rapidly and stoichiometrically by using DHS^ox^, following the reaction scheme of Equation (1).

(1) $$\text{R}\overset{{\text{DHS}}^{\text{ox}}}{⇀}1\text{S}\overset{{\text{DHS}}^{\text{ox}}}{⇀}2\text{S}\overset{{\text{DHS}}^{\text{ox}}}{⇀}3\text{S}\overset{{\text{DHS}}^{\text{ox}}}{⇀}4\text{S}$$

### 2.2. Long-Term Folding of HEL Using DHS^ox^

**Figure 3:**
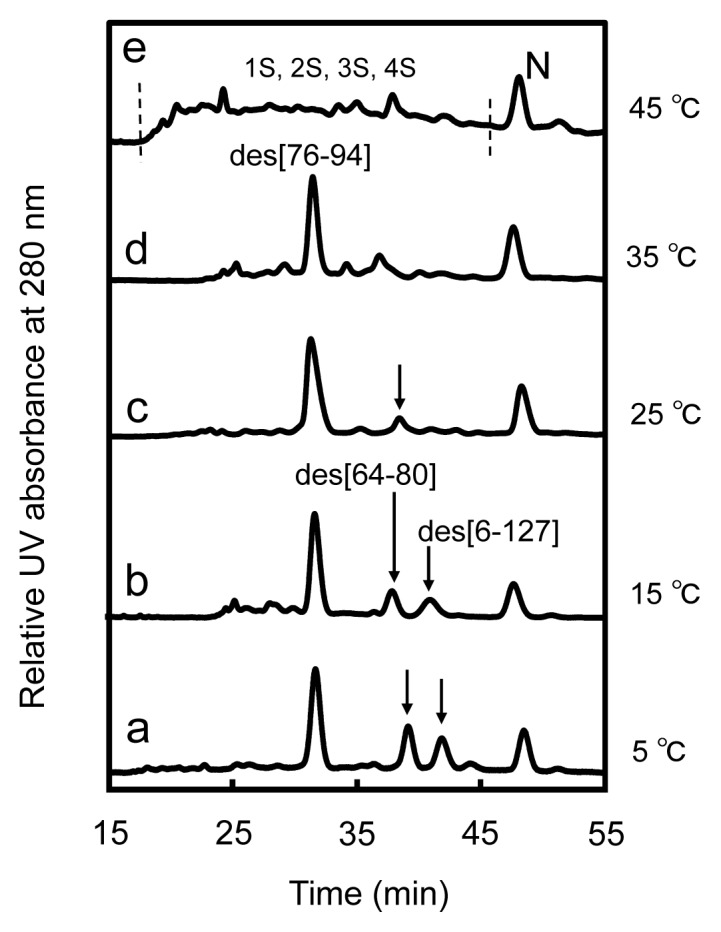
Reverse-phase HPLC chromatograms of the solutions (with AEMTS blocking) obtained from the long-term oxidative folding of HEL using three equivalents of DHS^ox^ at pH 8.0 for 300 min in the presence of 2 M urea. Reaction conditions were (**a**) [R]_0_ = [DHS^ox^]_0_/3 = 9.1 μM at 5 °C; (**b**) [R]_0_ = [DHS^ox^]_0_/3 = 9.8 μM at 15 °C; (**c**) [R]_0_ = [DHS^ox^]_0_/3 = 9.0 μM at 25 °C; (**d**) [R]_0_ = [DHS^ox^]_0_/3 = 9.2 μM at 35 °C; and (**e**) [R]_0_ = [DHS^ox^]_0_/3 = 10.5 μM at 45 °C. See the text for details of the HPLC analysis conditions.

The sample solutions obtained by the reaction of R with three equivalents of DHS^ox^ at pH 8.0 at 5, 15, 25, 35, and 45 °C were subsequently incubated in the presence of 2 M urea at the same temperature for long time (~22 h). The reverse-phase HPLC chromatograms of the sample solutions with an incubation time of 300 min are shown in Figure 3. At low temperatures (*i.e.*, 5 and 15 °C), the distinct peaks of des[76–94], des[64–80], and des[6–127] as well as N were observed (Figure 3a,b). These des intermediates, which are the metastable key folding intermediates of HEL, were unambiguously characterized by the thermolysin digestion (see Figures S2–S4 and Tables S2–S4). Since no DHS^ox^ was left in the solution and the intermolecular SS exchange reaction among oxidative folding intermediates must proceed much slower than the corresponding intramolecular process [11], the des intermediates of HEL should not be generated from 2S but from 3S via intramolecular SS rearrangement (*i.e.*, 3S→des). Indeed, the populations of the des intermediates were obviously decreased when two equivalents of DHS^ox^ were employed (see Figure S5). When similar long-term folding experiments were carried out at 25 °C, populations of des[64–80] and des[6–127] decreased significantly, while des[76–94] could be seen clearly in the HPLC chromatogram (Figure 3c). Although des[76–94] was still observed at 35 °C (Figure 3d), it disappeared at 45 °C (Figure 3e). The observation clearly demonstrated that each des intermediate has different thermodynamic stability. It is also notable that N can be gradually formed above 35 °C even though des[64–80] and des[6–127], which are the major precursors of N in the previous study (see Figure 1) [8], are no longer stable. In the long-term folding experiments (Figure 3), N must be formed from the des intermediates through slow intermolecular SS exchange reactions.

### 2.3. Equilibria between 3S and Des Intermediates

**Figure 4:**
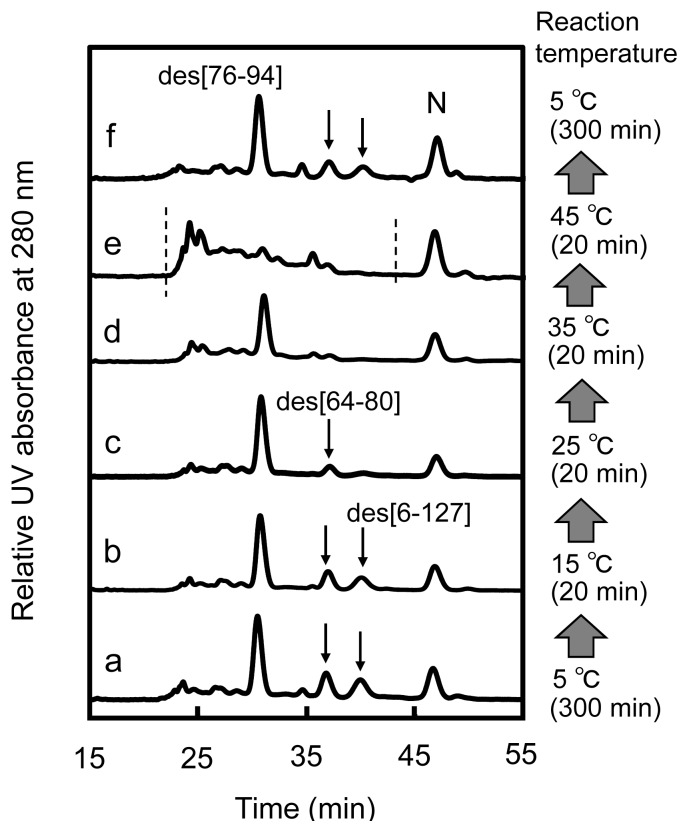
Reverse-phase HPLC chromatograms of the sample solutions (with AEMTS blocking) obtained from the temperature-jump experiment for the folding intermediates of HEL generated by the reaction of R with three equivalents of DHS^ox^ at pH 8.0 in the presence of 2 M urea. Reaction conditions were [R]_0_ = [DHS^ox^]_0_/3 = 9.8 μM. (**a**) Incubated at 5 °C for 300 min; (**b**) Incubated at 15 °C for 20 min after (**a**); (**c**) Incubated at 25 °C for 20 min after (**b**); (**d**) Incubated at 35 °C for 20 min after (**c**); (**e**) Incubated at 45 °C for 20 min after (**d**); (**f**) Incubated at 5 °C for 300 min after (**e**). See the text for details of the HPLC analysis conditions.

Reversible interconversion between the 3S and three des intermediates of HEL was confirmed by the temperature-jump experiment (Figure 4). When the folding solution obtained by the reaction of R with three equivalents of DHS^ox^ at 5 °C for 300 min was allowed to be maintained at 15 °C for 20 min, the populations of des intermediates did not change significantly. Subsequent treatment of the solution at 25 °C for 20 min caused an obvious decrease in the populations of des[64–80] and des[6–127]. Des[76–94], on the other hand, still remained with high population even at 35 °C. At 45 °C, the des intermediate disappeared, and a broad-band of the peaks, which would mainly correspond to the 3S intermediate ensemble, was observed. These population changes were almost completely retrieved when the solution was maintained again at 5 °C for 300 min. It is notable that the population of N did not change significantly after the temperature jump. This reasonably supports that the intermolecular SS exchange processes are very slow under the applied conditions. Thus, the presence of the equilibria between the 3S and des intermediates (Equations 2–4) was evident.

(2) 3S⇌des [76-94]

(3) 3S⇌des [64-80]

(4) 3S⇌des [6-127]

### 2.4. Reduction Pulse

**Figure 5:**
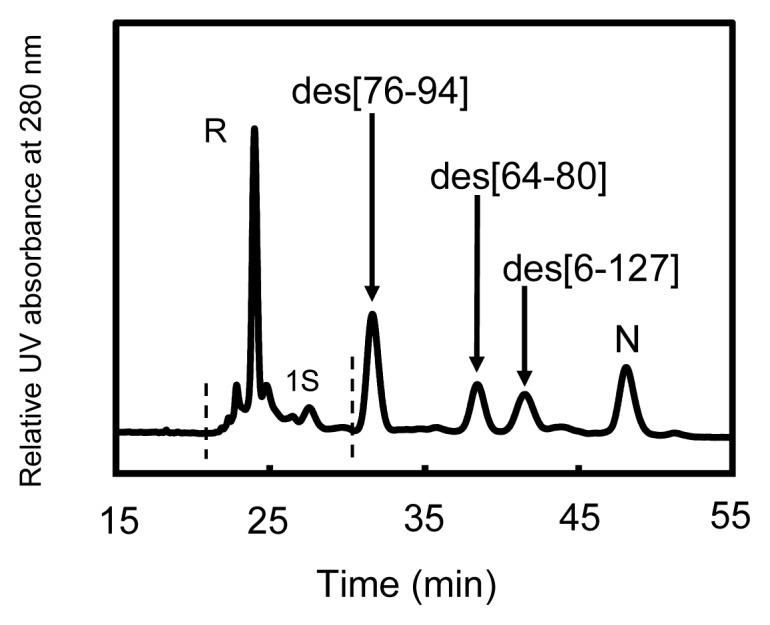
A reverse-phase HPLC chromatogram of the sample solution (with AEMTS blocking) obtained from the reduction pulse experiment of HEL using DTT^red^ as a reductant at 5 °C and pH 8.0 in the presence of 2 M urea. Folding conditions were [R]_0_ = [DHS^ox^]_0_/3 = 9.1 μM for 240 min. The reduction pulse conditions were 6 mM DTT^red^ for 3 min.

To estimate the exact populations of the des intermediates and N generated in the folding solutions, reduction pulse experiments [11,13] were performed. In the experiments, an excess amount of DTT^red^ was added before quenching the folding reaction by AEMTS so as to rapidly reduce the intermediates without rigid folded structure (*i.e*., 1S–4S) back to R (and/or 1S). Dobson and coworkers reported that the Cys6–Cys127 SS linkage of HEL can be reduced by 5 mM DTT^red^ at pH 7.8 [14,15]. Therefore, an investigation was made to optimize the reduction pulse conditions by changing the concentration of DTT^red^ and the reaction time. As a result, we found that the des intermediates having native-like structures and N can tolerate the reduction pulse under the conditions of 6 mM DTT^red^ for 3 min at 5–25 °C and 1 mM DTT^red^ for 2 min at 35 °C. The concentration of DTT^red^ was similar to the literature reduction condition [14,15]. However, the concentration of HEL was largely different between our experiments (~10 μM) and the literature (>1 mM). A typical HPLC chromatogram obtained from the reduction pulse experiment is shown in Figure 5. It is seen that the three des intermediates and N were isolated from the other species, *i.e.*, R and 1S. Similar results were obtained from the experiments under the other temperature conditions. Thus, exact populations of the des intermediates and N could be estimated. It should be noted that the populations thus obtained would involve systematic errors arising from the absorption change at 280 nm during the oxidative folding of HEL (~6%). However, the errors were not included in the following data analysis because only the relative populations of the des intermediates were employed to estimate the thermodynamic parameters.

**Figure 6:**
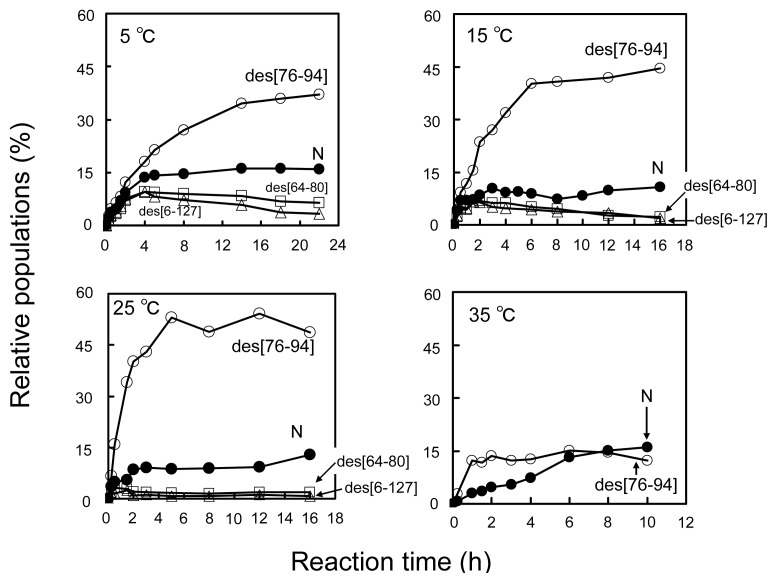
Population changes of the three des intermediates and N as a function of the reaction time at pH 8.0 in the presence of 2 M urea. The populations were estimated by the reduction pulse experiments. Folding conditions were [R]_0_ = [DHS^ox^]_0_/3 = 9.1 μM at 5 °C, [R]_0_ = [DHS^ox^]_0_/3 = 11.0 μM at 15 °C, [R]_0_ = [DHS^ox^]_0_/3 = 11.0 μM at 25 °C, and [R]_0_ = [DHS^ox^]_0_/3 = 9.2 μM at 35 °C. The reduction pulse conditions were 6 mM DTT^red^ for 3 min at 5 °C and 1 mM DTT^red^ for 2 min at 35 °C.

Figure 6 shows the plots of the populations of N and three des intermediates determined by application of the reduction pulse at 5, 15, 25, and 35 °C and pH 8.0 as a function of the folding time (15 min to 22 h). On the basis of the equilibria shown in Equations (2)–(4), the equilibrium constants (*K*_1–2_) between the des intermediates (Equations 5 and 6) as well as the relative free energies were subsequently calculated at each temperature. The results are summarized in Table 1.

(5) $$\text{des }[76-94]\overset{K_{1}}{⇌}\text{des }[64-80]$$

(6) $$\text{des }[76-94]\overset{K_{2}}{⇌}\text{des }[6-127]$$

The values of the equilibrium constants (*K*_1–2_) were determined by using the populations of des[76–94], des[64–80], and des[6–127] after a long period of the reaction time, when the pseudo equilibria attained. Free-energy differences of the des intermediates (Δ*G*_1–2_) were then estimated, except for those at 35 °C where des[64–80] and des[6–127] were not populated, probably due to the thermal denaturation. It is obvious that des[76–94] is the most stable among the three des intermediates in the range of temperature.

### 2.5. Conformational Changes during the Oxidative Folding of HEL

**Figure 7:**
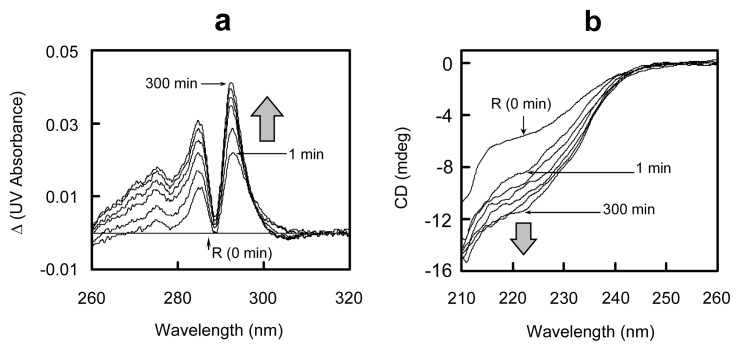
(**a**) Differential UV spectra and (**b**) CD spectra of the folding solutions of HEL at 5 °C and pH 8.0 in the presence of 2 M urea. Folding conditions were (**a**) [R]_0_ = [DHS^ox^]_0_/3 = 11.0 μM and (**b**) [R]_0_ = [DHS^ox^]_0_/3 = 10.6 μM.

To examine conformational changes during the oxidative folding of HEL, the UV and CD spectra were measured for the solutions. The obtained differential UV spectra and CD spectra are shown in Figure 7. The UV absorbance at 284 and 292 nm significantly increased within 1 min after initiation of the folding reaction (Figure 7a). The result indicated that the early SS formation reaction induces burying of the aromatic amino acid residues, such as tyrosine and tryptophan, into the molecule. Furthermore, the negative signal was observed around 222 nm in the CD spectrum of the folding solution within 1 min (Figure 7b), suggesting that some portion of α-helix is already formed by the SS formation reaction. By further incubation of the sample, both the UV and CD spectra gradually changed in the same directions, indicating maturation of the native conformation. The observed spectral changes were in accordance with the increase in the populations of the three des intermediates and N as shown in Figure 6.

### 2.6. Oxidation Pulse

To identify the effective precursors of N in the oxidative folding pathways of HEL, oxidation pulse experiments [11] were performed. After incubation of the reaction mixture of R and three equivalents of DHS^ox^ for 300 min, the generated SS intermediates were forced to be oxidized with 1.5 equivalents of DHS^ox^. The sample solutions obtained by the oxidation pulse were then treated by the reduction pulse at the same temperature in order to evaluate the population changes of N and the des intermediates.

**Figure 8:**
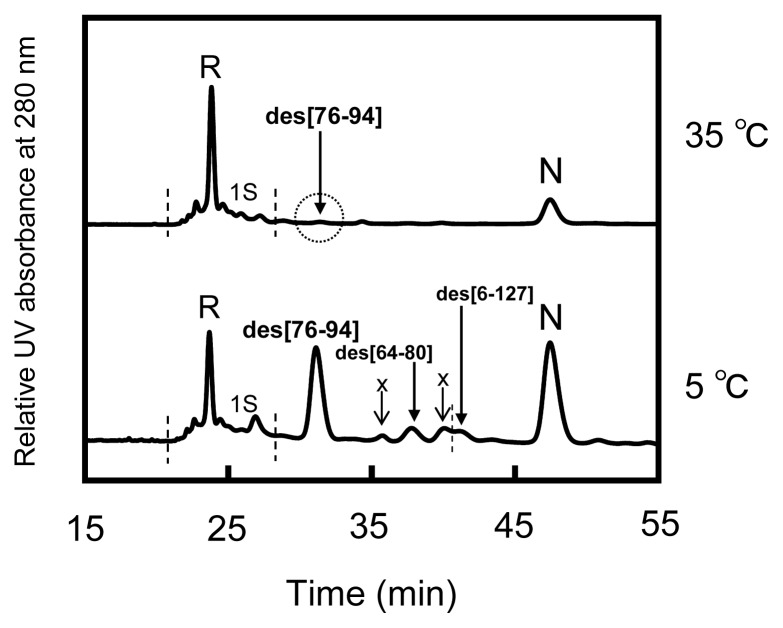
HPLC chromatograms of the sample solutions (with AEMTS blocking) obtained from the oxidation pulse experiments of HEL at 5 and 35 °C and pH 8.0 in the presence of 2 M urea. Folding conditions were [R]_0_ = [DHS^ox^]_0_/3 = 11.8 μM at 5 °C and [R]_0_ = [DHS^ox^]_0_/3 = 10.1 μM at 35 °C for 300 min. Oxidation pulse conditions were 1.5 eq DHS^ox^ for 3 min at 5 °C and for 20 min at 35 °C. The reduction pulse conditions were 6 mM DTT^red^ for 3 min at 5 °C and 1 mM DTT^red^ for 2 min at 35 °C. See the text for details of the HPLC analysis conditions.

**Figure 9:**
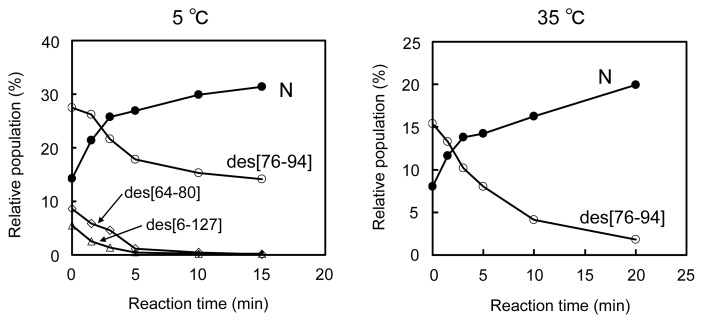
Population changes of des[76–94], des[64–80], des[6–127], and N as a function of the reaction time of the oxidation pulse (1.5 eq DHS^ox^) in the presence of 2 M urea at pH 8.0 at 5 and 35 °C. Folding conditions were [R]_0_ = [DHS^ox^]_0_/3 = 10.5 μM at 5 °C and [R]_0_ = [DHS^ox^]_0_/3 = 11.8 μM at 35 °C for 300 min. After the oxidation pulse, the same reduction pulse as Figure 8 was applied. See the text for details of the HPLC analysis conditions.

Figure 8 shows the HPLC chromatograms obtained from the oxidation pulse experiments at 5 °C (bottom panel) and 35 °C (top panel). At 5 °C, it is seen that the population of N obviously increased whereas those of des[64–80] and des[6–127] decreased, compared to the chromatogram shown in Figure 5. Indeed, when the population changes of the des intermediates were plotted as a function of a reaction time of oxidation pulse (Figure 9), it appeared that des[64–80] and des[6–127] are rapidly converted to N while the oxidation of des[76–94] is slow. Furthermore, Figure 9 shows that the increased ratio for N was roughly consistent with the sum of the decreased ratios of the des intermediates. The result clearly proved that des[64–80] and des[6–127] are major precursors of N, while des[76–94] is a minor precursor, in the oxidative folding pathways of HEL at 5 °C. At 35 °C, it was observed that des[76–94] is oxidized to N by the oxidation pulse (Figure 8). Since des[64–80] and des[6–127] are not populated at 35 °C (see Figure 6) and the population changes of des[76–94] and N observed in the oxidation pulse experiment seem to be mutually complementary (Figure 9), it is likely that N is dominantly formed through des[76–94] at 35 °C.

## 3. Discussion

### 3.1. Precursors of the Des Intermediates

In this study, oxidative folding of HEL was carried out by using DHS^ox^ as a strong oxidant, which enables rapid and stoichiometric SS formation in polythiol substrates [10]. As a result, the three des intermediates, *i.e.*, des[76–94], des[64–80], and des[6–127], which have previously been characterized as the key intermediates on the folding pathways toward N [7,8], were observed. Since the populations of these des intermediates were significantly decreased when two equivalents of DHS^ox^ were reacted with R (see Figure S5), the des intermediates should be mainly formed from the 3S intermediate ensemble through SS rearrangement. In the previous pathways (Figure 1), however, it was suggested that 2S is directly oxidized to the des intermediates. This discrepancy would be due to the competition between the oxidation from 2S to 3S and the isomerization from 3S to the des intermediates when GSSG is used as an oxidant. Such SS-based folding reagents have low oxidation abilities, hence 3S cannot be populated in the folding solution. In contrast, when a strong oxidant, such as DHS^ox^, is applied, SS formation (*i.e.*, oxidation) proceeds much faster than SS rearrangement. This allows accumulation of 3S prior to the rearrangement to the des intermediates [9,11].

The rearrangement to the des intermediates is the process that accompanies formation of the stable native conformation of HEL [8]. This was confirmed in this study by a couple of experimental observations. First, in the reduction pulse experiments (Figure 5) the early SS intermediates, 1S–4S, were rapidly reduced with DTT^red^, while the des intermediates survived from the reduction pulse, suggesting that rigid structures were generated during the SS rearrangement from 3S to the des intermediates. Second, the UV and CD spectral changes during the rearrangement process (1–300 min) (Figure 7) clearly showed maturation of the native tertiary and secondary structures. Thus, it was reconfirmed that the generation of the des intermediates is the most important process to obtain the stable native conformation on the oxidative folding pathways of HEL.

Although the 1S–4S intermediates do not have rigid structures, they would statistically have the native-like structures to some extent [16,17]. As seen in Figure 7, new peaks appear at 292, 285, and 275 nm in the UV spectrum and at around 222 nm in the CD spectrum within 1 min after the addition of DHS^ox^ to R. The positive increase of the UV absorption band at 292 nm suggests the hydrophobic collapse, which brings the tryptophan residues buried during the oxidation of R [16,17]. The negative increase of the CD spectrum at 222 nm indicates formation of the helical secondary structures in 1S–4S. It was previously demonstrated that 1SS and 2SS-variants of HEL having one and two native SS linkages, respectively, have native-like secondary structures [18–24]. This is in accord with our observations because the SS intermediate cocktail formed within 1 min should contain the native SS linkages in some ratios. Such an intermediate cocktail would conformationally resemble the molten globule of HEL observed in the early stage of the SS-intact folding [25]. In the case of RNase A, the similar conformational resemblance was observed between the mixture of 1S–4S intermediates and the transient state generated in the burst phase of the SS-intact folding [12]. Thus, association of the SS-intact folding processes with those of the SS-reduced folding would be possible at least for these two typical proteins.

### 3.2. Relative Stability of the Des Intermediates

The populations of the three des intermediates of HEL should reflect the relative thermodynamic stability at the applied temperature because they can be converted to each other through 3S as shown in Figure 4. According to the HPLC chromatograms obtained with a long reaction time (300 min) (Figure 3), the heat denaturation temperatures (*T*_m_) of des[76–94], des[64–80], and des[6–127] would be around 35–45, 25–35, and 15–25 °C, respectively, at pH 8.0 in the presence of 2 M urea. The estimation is reasonable in light of the *T*_m_ values reported for the corresponding 3SS variants of HEL, *i.e.*, C76A/C94A, C64A/C80A, and C6S/C127A, at pH 3.0 [26]. Des[76–94] is most stable in the range of the applied temperatures, indicating that the Cys76–Cys94 SS linkage, which connects the α- and β-domains of HEL, contributes less to the thermodynamic stability of the native conformation than the other two SS linkages, *i.e.*, Cys64–Cys80 and Cys6–Cys127. This would be in agreement with the previous observation that these SS linkages play significant roles in the kinetic and thermodynamic stabilization of the SS intermediates formed in the early folding stage [16,27,28].

Another des intermediate, des[30–115], could not be observed during the oxidative folding of HEL, although the corresponding 3SS variant of HEL, C30A/C115A, was reported to have native-like folded structure [29] with the *T*_m_ of 35 °C [26] at pH 3.0. This would suggest that formation of des[30–115] is prohibited kinetically due to the predominant formation of the Cys30–Cys115 SS linkage, which is located in the hydrophobic core of native HEL, in the 1S–4S intermediate cocktail.

### 3.3. Switching the Major Pathways toward N by Temperature Control

In previous studies, the oxidation reactions of des[64–80] and des[6–127] were assigned to the major folding pathways toward N at pH 8.5 and 20 °C in the presence of 2 M urea [8]. The same processes were clearly observed in this study at pH 8.0 and 5 °C in the presence of 2 M urea (Figure 8). At temperatures up to 25 °C, these pathways would be major routes to N because des[64–80] and des[6–127] can be populated. Meanwhile, des[76–94] would have robust conformation keeping the free Cys94 residue inside of the native-like fold [7]. Thus, the SS linkage between the Cys76 and Cys94 residues cannot easily approach to each other. This is in accord with the observation that although des[76–94] was populated in a large amount, the formation of N was very slow in the long-term folding experiments (Figure 6). Thus, the des[76–94] to N route should be significantly hindered at low temperatures. However, the direct oxidation route from des[76–94] to N should not be completely prohibited because the formation of N can be observed at 5 °C in the oxidation pulse experiment (Figure 9) even after des[64–80] and des[6–127] vanished (>5 min). On the other hand, the major pathway of HEL at 35 °C was found to be significantly different since des[64–80] and des[6–127] are no more stable at the temperature (Figures 3d and 4d). The gradual formation of N seen in Figure 6d is also significantly different from the behavior observed at the other temperatures, suggesting the major folding pathway to N has changed at 35 °C. In the oxidation pulse experiment at 35 °C (Figure 9), a roughly quantitative conversion from des[76–94] to N was observed. Since the direct oxidation of des[76–94] to N is possible at 5 °C, as discussed above, the same route must exist at higher temperatures. At 35 °C, the conformation of des[76–94] would become a little flexible to allow the acceleration of the Cys76–Cys94 SS linkage formation. There might be another possibility that before the oxidation to N, des[76–94] would be denatured and converted to des[64–80] or des[6–127] as observed in the oxidative folding of HEL in the presence of PDI [8]. However, this route is unlikely because des[64–80] and des[6–127] must be present in only small amounts in the scrambled 3S intermediate ensemble at 35 °C and, therefore, most of 3S should be rapidly oxidized to misfolded 4S under the oxidation pulse conditions, if it was possible. Thus, the major pathways of HEL can be switched from one to the other by temperature control.

Switching the major folding pathways of proteins by temperature control would be an important phenomenon, as folding pathways for most SS-containing proteins have usually been studied in a limited temperature range. Our results would be the first example to clearly show that the oxidative folding pathways can change from one to the other depending on the temperature. Narayan *et al*. [30] previously summarized the patterns of the final oxidation processes in the oxidative protein folding pathways. According to their definitions, pathway I is the direct generation of N from an unfolded des intermediate. This pathway would be effective at 45 °C in the case of HEL because N was formed in spite of the absence of any des intermediates (see Figure 3e). Pathway II is the oxidation of a stable des intermediate to N. This pathway corresponds to the major pathways of HEL observed at the temperatures below 35 °C. At 5–25 °C, des[64–80] and des[6–127] are mainly oxidized to N, while oxidation of another des intermediate, des[76–94], is a minor pathway to N. At 35 °C, however, the former des intermediates are denatured. Instead, the process from des[76–94] to N became effective. Pathway III is a metastable des pathway, in which a metastable des intermediate is transformed to another stable des intermediate and then oxidized to N. Pathway IV is a metastable dead-end pathway. These pathways were not observed in the oxidative folding of HEL.

## 4. Experimental Section

### 4.1. Materials

HEL was purchased from Sigma Aldrich Japan and used without purification. DHS^ox^[31] and AEMTS [32] were synthesized according to the literature methods. All other reagents were commercially available and used without further purification.

### 4.2. Preparation of Reduced HEL (R)

The experimental procedure previously described [7,33] was followed with slight modification. To a solution of HEL (12 mg) dissolved in 0.7 mL of a 100 mM Tris–HCl/1 mM EDTA buffer solution at pH 8.0 containing 8 M urea as a denaturant was added an excess amount of DTT^red^ (12 mg). The reaction mixture was incubated at 40 °C for 120 min. Resulting R solution was diluted with 1.3 mL of 0.1 M acetic acid and purified by passing through the column packed with Sephadex G25 resin, which was equilibrated with a 0.1 M acetic acid (*ca.* pH 3.0). After lyophilization of the desalted R solution, the resulting white powder material of R was stored at −30 °C.

To prepare R solutions at pH 8.0, 0.5–1.0 mg of white powder materials of R was dissolved in 1.0 mL of a 100 mM Tris–HCl/1 mM EDTA buffer solution at pH 8.0 containing 8 M urea. The stock solution was diluted two-fold with the same pH buffer solution without urea. The concentration of R was determined by UV absorbance at 280 nm using the molar extinction coefficient (ɛ_280_ = 33,890 M^−1^cm^−1^[34]). The R solution was immediately used in the following folding experiments. Dissolved oxygen was removed from the buffer solutions by purging nitrogen gas in order to avoid air oxidation of the free thiol groups.

### 4.3. Oxidative Folding Experiment

A DHS^ox^ solution in 100 mM Tris–HCl/1 mM EDTA buffer at pH 8.0 containing 4 M urea was prepared so that the concentration of DHS^ox^ was one-, two-, three-, or four-fold with respect to R. The R and DHS^ox^ solutions were maintained at 5.0, 15.0, 25.0, 35.0, or 45.0 ± 0.1 °C in a dry thermo bath. 100 μL of the R solution was then manually added with 100 μL of the DHS^ox^ solution in a 1.5 mL micro centrifuge tube. The mixture was incubated for 1 min at (5.0–45.0) ± 0.1 °C and was diluted two-fold with the same buffer solution without urea. The sample solution was subsequently incubated at each temperature for 1 min–22 h.

Three sample solutions with a different post-oxidation treatment were prepared at each reaction time. The first sample solution was added with 300 μL of an aqueous AEMTS solution (10 mg/mL) to quench SS rearrangement reactions (without a redox pulse). For the second sample solution, a reduction pulse was applied according to the literature [11,13]. Before quenching the reactions by addition of AEMTS, 100 μL of a 100 mM Tris–HCl/1 mM EDTA buffer solution at pH 8.0 containing DTT^red^ and 2 M urea was added to the sample solution (a reduction pulse). After 2–3 min at the same temperature, the reaction was quenched with 420 μL of the aqueous AEMTS solution. For the third sample solution, an oxidation pulse was applied [11]. Before the AEMTS quenching, 100 μL of a 100 mM Tris–HCl/1 mM EDTA buffer solution at pH 8.0 containing 1.5 equivalents of DHS^ox^ with respect to R and 2 M urea was added to the sample solution (an oxidation pulse). After incubation for 1.5–20 min at the same temperature, the sample solution was treated with DTT^red^ for 2–3 min and then the reaction was quenched with 400 μL of the AEMTS solution. The collected sample solutions were acidified to pH 3–4 with 10 μL of acetic acid and stored at −30 °C.

To investigate formation of the native structures during the oxidative folding reaction, the UV and CD spectra were measured by using a Shimadzu UV-1700 spectrophotometer (Shimadzu Corporation, Kyoto, Japan) and a Jasco J-820 spectrophotometer (Jasco Corporation, Hachioji, Japan), respectively, in parallel to the above long-term oxidative folding experiment. To measure the UV spectrum, 300 μL each of the R and DHS^ox^ solutions was mixed and incubated at 1 min for at 5 °C. The solution was then diluted with 600 μL of the same buffer solution without urea and poured into a quartz cell having 1 cm light path length, which was set in the UV cell holder thermostated at 5.0 ± 0.1 °C. Similarly, to measure the CD spectrum, the folding solution prepared by the same procedure as described above was poured into a quartz cell having 1 mm light path length, which was set in the CD cell holder thermostated at 5.0 ± 0.1 °C.

### 4.4. Temperature-Jump Experiments

R and DHS^ox^ solutions with the concentration ratio of 1:3 were cooled at 5.0 ± 0.1 °C in a thermostated water bath. 3 mL of the R solution was manually added with 3 mL of the DHS^ox^ solution in a 15 mL centrifuge tube. The mixture containing 4 M urea was incubated for 1 min at 5.0 ± 0.1 °C in a thermostated water bath and was diluted two-fold with the same buffer solution without urea. This sample solution was subsequently incubated at 5.0 °C for 300 min. An aliquot (400 μL) of the reaction solution was taken out and reacted with 200 μL of an aqueous AEMTS solution (10 mg/mL) to quench the reaction. The temperature of the water bath was then increased to 15.0 ± 0.1 °C. After 20 min, an aliquot (400 μL) of the reaction solution was taken out and reacted with 200 μL of the AEMTS solution. Similarly, the temperature was further increased at 25.0, 35.0, and 45.0 °C each for 20 min and then decreased at 5.0 °C for 300 min. At each temperature an aliquot of the reaction solution was reacted with AEMTS. The collected sample solutions were acidified and stored at −30 °C.

### 4.5. HPLC Analysis

The sample solutions obtained from the above folding experiments were thawed and added with 1.2 mL of 0.1 M acetic acid. The diluted sample solutions were subsequently desalted by using a Sephadex G25 column equilibrated with 0.1 M acetic acid. After lyophilization of the desalted solutions, the obtained white powder materials of the folding intermediates were dissolved in 1.1 mL of 0.1% TFA in water. The sample solutions were analyzed by HPLC equipped with a 1 mL sample solution loop and a Tosoh TSKgel ODS-100V 4.6 × 150 mm reverse phase column. The column was equilibrated with 80:20 (*v*/*v*) mixture of 0.1% TFA in water (eluent A) and 0.1% TFA in acetonitrile (eluent B) at a flow rate of 0.5 mL/min. After injection of the sample solution, a solvent gradient (a ratio of eluent B linearly increased from 20% to 34% in 0–14 min, from 34% to 35% in 14–30 min, from 35% to 38% in 30–60 min, and from 38% to 100% in 60–62 min) was applied. The folding intermediates were detected by UV absorption at 280 nm. The recorded signals were integrated and analyzed by using a Shimadzu LC solution software (version 1.03 SP5; Shimadzu Corporation, Kyoto, Japan).

### 4.6. Characterization of the Folding Intermediates

The molecular mass of the intermediates obtained by oxidation of R with one- to four-fold DHS^ox^ for 1 min was measured on a Jeol JMS-T100LP mass spectrometer operated in the ESI(+) mode connected to an Agilent 1200 series HPLC system. Each SS ensemble had a different mass number due to modification of the free SH groups with AEMTS. Ellman’s assay [35] was also performed for the same intermediate mixtures to confirm the stoichiometric oxidation with DHS^ox^. On the other hand, the positions of the SS linkages in the isolated three des intermediates were determined according to the enzymatic digestion experiment reported previously [36]: The AEMTS-blocked and lyophilized des intermediate dissolved in 50 mM *N*-ethyl morpholine acetate buffer at pH 6.4 (115 μg/mL) was digested by thermolysin (40 μg/mL) at 47 °C for 14 h. After quenching with a 10% TFA aqueous solution, 15 μL of the sample solution containing the peptide fragments was injected to the LC-MS instrument equipped a Tosoh TSKgel ODS-100V 4.6 × 150 mm reverse phase column. A binary solution gradient of 0.1% TFA or formic acid in water (eluent A) and 0.1% TFA or formic acid in acetonitrile (eluent B) (a ratio of eluent B linearly increased from 0 to 10% in 0–30 min, from 10% to 42% in 30–55 min, and from 42% to 100% in 55–100 min) was applied with a flow rate of 1.0 mL/min.

## 5. Conclusions

**Figure 10:**
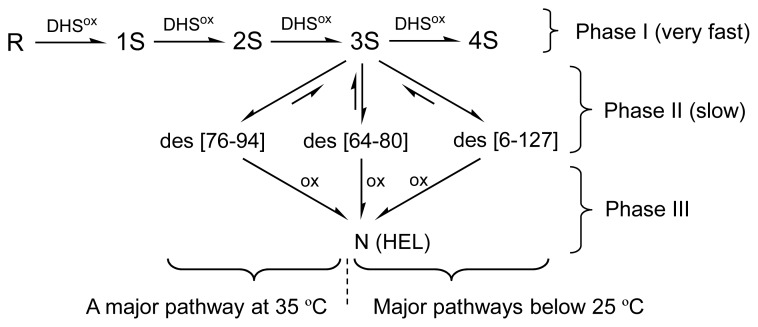
The oxidative folding pathways of HEL characterized by using DHS^ox^ at pH 8.0 in the presence of 2 M urea.

By using DHS^ox^ as a strong oxidant, oxidative folding pathways of HEL were reinvestigated in this study. As a result, the new folding pathways have been clearly characterized as illustrated in Figure 10.

At 5–25 °C, R is rapidly oxidized with DHS^ox^ (within 1 min) to produce a random SS intermediate cocktail (*i.e*., 1S–4S) without any stable structures, accompanying hydrophobic collapse and formation of the weak native-like secondary structures to some extent (phase I). Subsequently, 3S is slowly converted to the three des intermediates, *i.e.*, des[76–94], des[64–80], and des[6–127], through reversible SS rearrangement (phase II). This folding phase corresponds to the main conformational folding processes to acquire the stable native structures. In the final step (phase III), all the three des intermediates can be oxidized to N, but the oxidation of des[64–80] and des[6–127] constitutes major routes to N. The pathways are in agreement with the previous ones (Figure 1), expect for the precursor of the des intermediates. The discrepancy would be due to the difference in the oxidizing ability of the oxidants employed in the folding experiments. A strong oxidant, such as DHS^ox^, allows completion of the SS formation process before the SS rearrangement takes place, while a weak oxidant, such as GSSG, allows competition of these processes.

At 35 °C, on the other hand, the major pathway toward N is switched to the oxidation of des[76–94] to N, which is a hindered pathway at lower temperatures. The switching is not only due to the loss of conformational stability for des[64–80] and des[6–127] but also due to the gain of conformational flexibility for des[76–94] at 35 °C. This would be the first example to clearly show that the oxidative folding pathways can be changed from one to the other by temperature control. At 45 °C, N would be formed through the unfolded des intermediates. To conclude, our results indicate that the des[76–94] to N route should be dominant under physiological temperature conditions. According to the remarkable temperature effects on the oxidative folding pathways of HEL, it is recommended to reinvestigate the folding pathways of other proteins at temperatures close to physiological conditions.

## Supplementary Information



## Figures and Tables

**Table 1:** Thermodynamic parameters for the equilibria between the three des intermediates of HEL at pH 8.0 in the presence of 2 M urea.

Temperature (°C)	des[76–94]_∞_^a^ (%)	des[64–80]_∞_^a^ (%)	des[6–127]_∞_^a^ (%)	Equilibria	*K*^b^	Δ*G*^c^ (kcal/mol)
5	36 (±1)	7 (±1)	4 (±1)	des[76–94] ⇌ des[64–80]	0.19 (±0.03)	0.9 (±0.1)
				des[76–94] ⇌ des[6–127]	0.11 (±0.03)	1.2 (±0.1)
15	42 (±3)	4 (±3)	3 (±2)	des[76–94] ⇌ des[64–80]	0.10 (±0.07)	1.3 (±0.7)
				des[76–94] ⇌ des[6–127]	0.07 (±0.05)	1.5 (±0.7)
25	51 (±3)	2 (±1)	1 (±1)	des[76–94] ⇌ des[64–80]	0.04 (±0.02)	1.9 (±0.4)
				des[76–94] ⇌ des[6–127]	0.02 (±0.02)	2.3 (±0.4)
35	13 (±2)	NG d	NG	des[76–94] ⇌ des[64–80]	ND e	ND
				des[76–94] ⇌ des[6–127]	ND	ND

a Populations of the des intermediates in the total protein after the reaction time of 14, 6, 5, and 2 h at 5, 15, 25, and 35 °C, respectively.

b Equilibrium constants.

c Free energy differences between the des intermediates.

d Not generated.

e Not determined. (not italic)

## Abbreviations

1S, 2S, 3S, and 4S: ensembles of folding intermediates of HEL with one, two, three, and four SS linkages, respectively
AEMTS: 2-aminoethyl methanethiosulfonate
CD: circular dichroism
des[76–94], des[6–127], and des[64–80]: structured 3S intermediates of HEL having three native SS bonds but lacking one native SS bond specified
DHS^ox^: *trans*-3,4-dihydroxyselenolane oxide
DTT^red^: dithiothreitol
EDTA: ethylenediaminetetraacetic acid
ESI: electron spray ionization
GSH: glutathione
GSSG: oxidized glutathione
HEL: hen egg white lysozyme
HPLC: high performance liquid chromatography
N: native HEL
R: reduced HEL
RNase A: ribonuclease A
SH: thiol
SS: disulfide
TFA: trifluoroacetic acid
UV: ultraviolet.
