# Supplementary material for: Reinvestigation of the Oxidative Folding Pathways of Hen Egg White Lysozyme: Switching of the Major Pathways by Temperature Control
Source: Int J Mol Sci. 2013 Jun 26;14(7):13194–212. doi: 10.3390/ijms140713194 (PMC3742182; doi:10.3390/ijms140713194)
Supplement: Supplementary file 1 [file ijms-14-13194-s001.pdf]

Supplementary Information

1. Ellman's Assay

Figure S1. Relative concentration of SH groups estimated by Ellman's assay for the sample solutions (without AEMTS blocking) obtained by the oxidation of R with one, two, three, and four equivalents of DHS^{ox} for 1 min at pH 8.0 and 25 °C in the presence of 4 M urea.

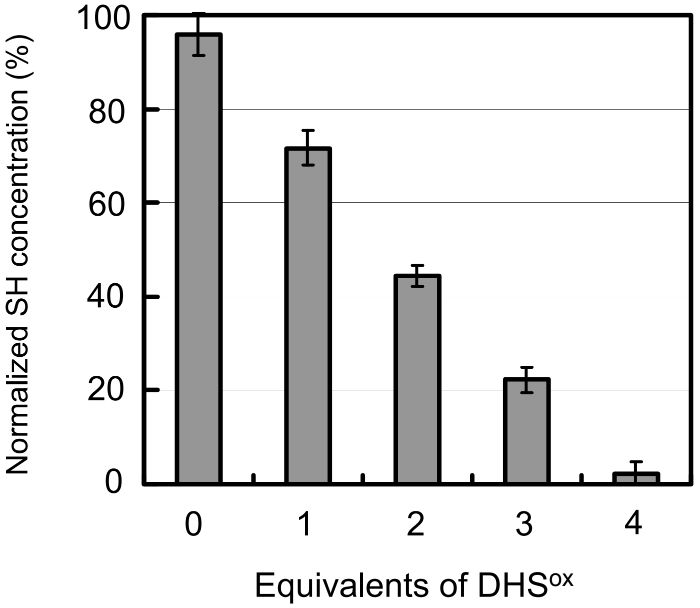

2. Summary of Mass Numbers Obtained by ESI-TOF-MS Analysis

Table S1. Expected and observed mass numbers for R, 1S, 2S, 3S, 4S, des[76–94], des[64–80], des[6–127], and N with AEMTS blocking.

Species	Expected Mass (*m*/*z*)	Obsd. Mass (*m*/*z*)	Charge	Calcd. Mass (*m*/*z*) [a]
R [b]	14915	1658.19924	+9	14915
R [c]	14915	1492.497	+10	14915
1S [c]	14763	1477.355	+10	14764
2S [c]	14611	1461.785	+10	14608
3S [c]	14459	1446.813	+10	14458
4S [c]	14307	1431.619	+10	14306
des[76–94] [b]	14459	2892.85095	+5	14459
des[64–80] [b]	14459	2892.83217	+5	14459
des[6–127] [b]	14459	2892.77762	+5	14459
N [b]	14307	2862.43854	+5	14307

[a] The calculated mass numbers were obtained from the observed mass numbers using the equation, (Calcd. Mass) = (Obsd. Mass) x (Charge) – (Charge), based on the reaction, $M + nH^{+} \rightarrow MH_{n}^{n+}$.
[b] The species separated by reverse-phase HPLC analysis. [c] The species observed in Figure 2.

3. Mass Chromatograms for Thermolysin-Digested des[76–94], des[64–80] and des[6–127] Intermediates

Figure S2. Mass chromatogram of the peptide fragments obtained from des[76–94] by thermolysin digestion. The peak numbers correspond to those in Table S2. Non-labeled peaks could not been assigned reasonably. 0.1% TFA in water and 0.1% TFA in acetonitrile were employed as eluents.

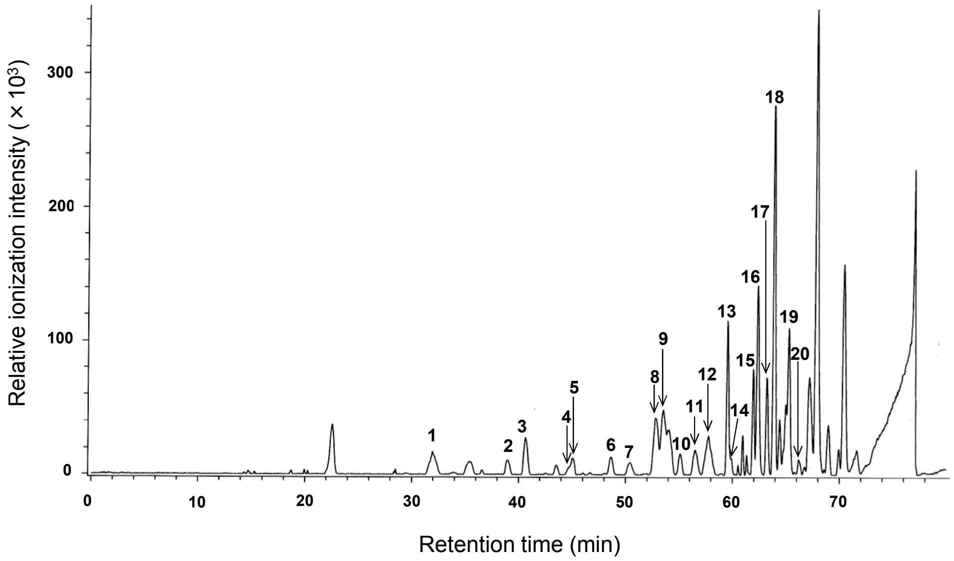

Table S2. Structure assignments for the thermolysin-digested fragments of des[76–94]. [a]

Peak No. [b]	Retention time (min)	Expected mass	Observed mass	Peptide Fragment	Cys or SS pairing
1	31.99	690.32	691.50 (+1)	105–110	No Cys
2	38.97	505.53	504.27 (+1)	86–90	No Cys
3	40.67	574.59	575.29 (+1)	42–46	No Cys
4	44.74	520.60	521.30 (+1)	105–108	No Cys
5	45.09	644.68	644.35 (+1)	20–24	No Cys
6	48.67	538.22	539.22 (+1)	74–77	Cys76
7	50.46	719.68	721.35 (+1)<br>361.17 (+2)	43–48	No Cys
8	52.84	627.75	628.35 (+1)	12–16	NoCys
9	53.54	488.53	489.28 (+1)	58–61	NoCys
10	55.10	698.84	699.39 (+1)	11–16	NoCys
11	56.50	505.53	505.30 (+1)	103–107	No Cys
12	57.78	394.42	395.20 (+1)	20–22	No Cys
13	59.53	660.66	661.33 (+1)	98–104	No Cys
14	59.89	922.93	922.42 (+1)	69–77	Cys76
15	61.92	905.96	906.42 (+1)	83–91	No Cys
16	62.36	736.38	767.38 (+1)	92–97	Cys94
17	63.22	1208.44	1208.61 (+1)<br>604.81 (+2)	62–66, 67–73	Cys30–Cys115

Table S2. *Cont.*

Peak No. [b]	Retention time (min)	Expected mass	Observed mass	Peptide Fragment	Cys or SS pairing
18	63.86	1178.42	1179.64 (+1)<br>589.81 (+2)	4–7, 124–129	Cys6–Cys127
19	65.25	1050.07	1049.49 (+1)	62–66, 78–80	Cys64–Cys80
20	66.20	1324.66	1325.69 (+1)<br>663.35 (+2)	3–7, 124–129	Cys6–Cys127

[a] Detailed experimental conditions are described in the text. [b] The number corresponds of the peak number in Figure S2.

Table S3. Structure assignments for the thermolysin-digested fragments of des[64–80]. [a]

Peak No.	Retention time (min)	Expected mass	Observed mass	Peptide Fragment	Cys or SS pairing
1	1.99	374.43	375.67 (+1)	105–110	No Cys
2	2.33	574.59	574.46 (+1)	42–46	No Cys
3	2.48	432.42	433.61 (+1)	39–42	No Cys
4	3.39	505.53	504.58 (+1)	103–107	No Cys
5	4.63	488.54	488.57 (+1)	25–28	No Cys
6	5.05	394.42	394.60 (+1)	20–22	No Cys
7	8.15	533.56	532.48 (+1)	83–87	No Cys
8	13.21	660.66	660.47 (+1)	98–104	NoCys
9	35.71	669.61	668.49 (+1)	63–67	Cys64
10	39.22	977.02	976.05 (+1)<br>488.53 (+2)	98–107	NoCys
11	54.32	627.75	627.58 (+1)	12–16	No Cys
12	55.34	1007.97	1008.92 (+1)	75–77, 92–97	Cys76–Cys94
13	55.34	1055.50	1052.86 (+1)	3–7, 126–129	Cys6–Cys127

[a] Detailed experimental conditions are described in the text. [b] The number corresponds of the peak number in Figure S3.

Figure S3. Mass chromatogram of the peptide fragments obtained from des[64–80] by thermolysin digestion. The peak numbers correspond to those in Table S3. Non-labeled peaks could not been assigned reasonably. 0.1% formic acid in water and 0.1% formic acid in acetonitrile were employed as eluents.

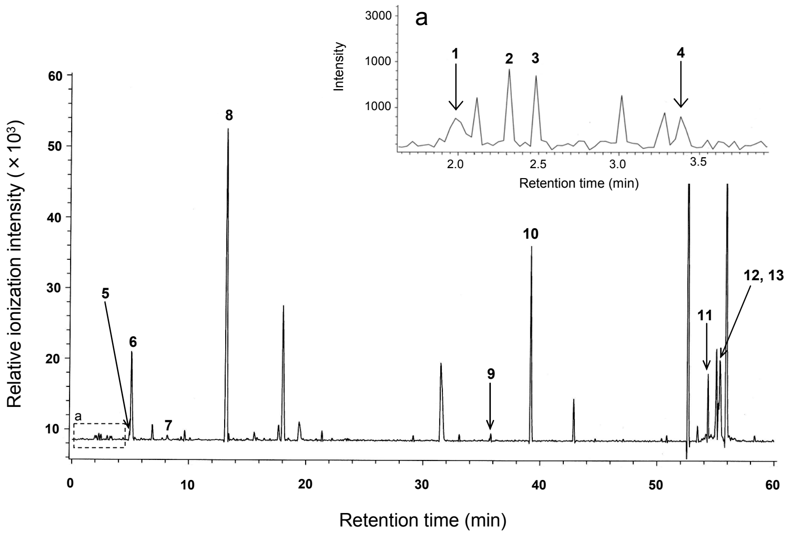

Figure S4. Mass chromatogram of the peptide fragments obtained from des[6–127] by thermolysin digestion. The peak numbers correspond to those in Table S4. Non-labeled peaks could not been assigned reasonably. 0.1% formic acid in water and 0.1% formic acid in acetonitrile were employed as eluents.

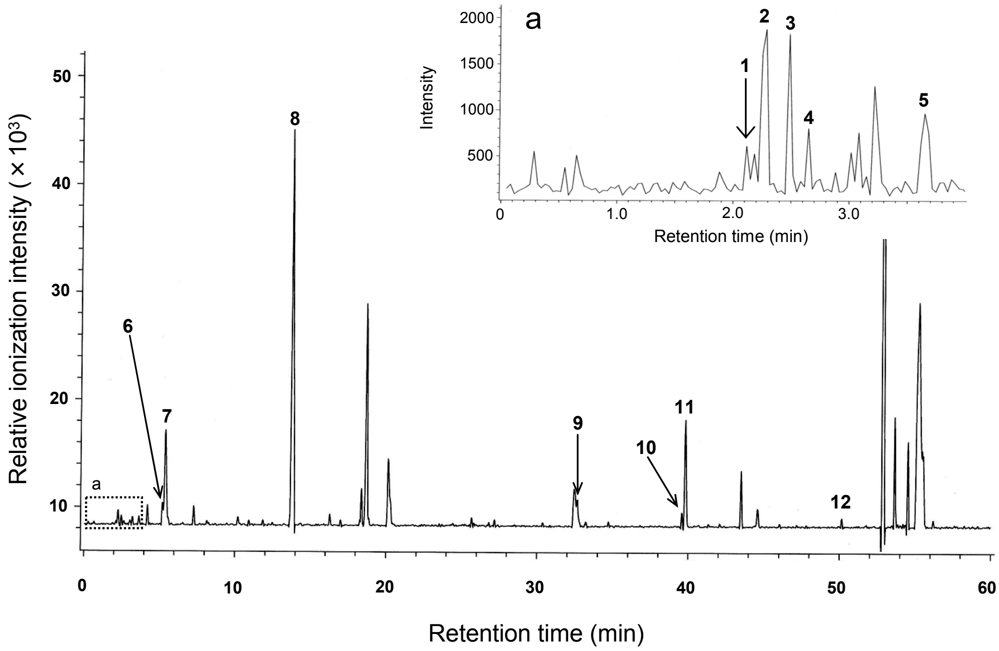

Table S4. Structure assignments for the thermolysin-digested fragments of des[6–127]. [a]

Peak No.	Retention time (min)	Expected mass	Observed mass	Peptide Fragment	Cys or SS pairing
1	2.12	374.43	376.27	108–110	No Cys
2	2.28	539.18	539.30	4–7	Cys6
3	2.49	574.59	575.38	42–46	No Cys
4	2.65	432.42	433.61	39–42	No Cys
5	3.66	505.53	505.38	103–107	No Cys
6	5.21	488.54	489.35	25–28	No Cys
7	5.41	394.42	395.24	20–22	No Cys
8	13.77	660.66	661.42	98–104	No Cys
9	32.48	1078.72	1077.15	63–68, 78–80	Cys64–Cys80
10	39.58	785.80	787.44	2–7	Cys6
11	39.81	977.02	977.65(+1) 489.33(+2)	98–107	No Cys
12	50.14	893.91	893.21	75-76, 92-97	Cys76–Cys94

[a] Detailed experimental conditions are described in the text. [b] The number corresponds of the peak number in Figure S4.

4. HPLC Chromatogram Obtained by Oxidative Folding at 5 °C using 2 Equivalents of DHS^{ox}

Figure S5. Reverse-phase HPLC chromatogram of the solution (with AEMTS blocking) obtained by the reduction pulse experiments of HEL using DTT^{red} as a reductant at 5 °C and pH 8.0 in the presence of 2 M urea. Folding conditions was $[R]_0 = [DHS^{ox}]_0/2 = 53.6$ μM for 300 min. The reduction pulse condition was 6 mM DTT^{red} for 3 min at 5 °C.

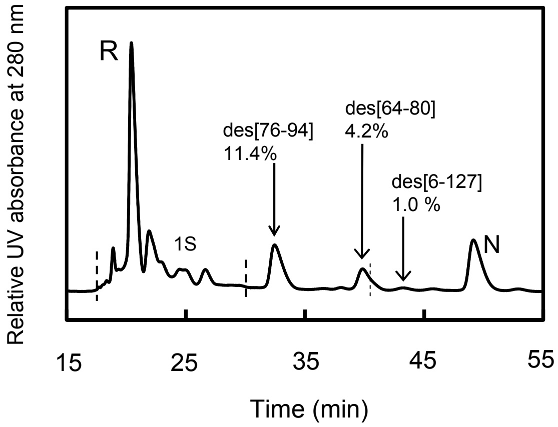

© 2013 by the authors; licensee MDPI, Basel, Switzerland. This article is an open access article distributed under the terms and conditions of the Creative Commons Attribution license (http://creativecommons.org/licenses/by/3.0/).
